# Opening wedge high tibial osteotomy yields comparable to superior outcomes to unicompartmental knee arthroplasty at 2 years of follow‐up in patients suffering from Ahlbäck III knee osteoarthritis: A propensity score‐matched analysis

**DOI:** 10.1002/jeo2.70105

**Published:** 2024-12-02

**Authors:** Shintaro Onishi, Christophe Jacquet, Hiroshi Nakayama, Jean Noël Argenson, Matthieu Ollivier

**Affiliations:** ^1^ Aix‐Marseille University, APHM, CNRS, ISM, Sainte‐Marguerite Hospital Institute for Locomotion Marseille France; ^2^ Department of Orthopedic Surgery Hyogo Medical University Nishinomiya Japan

**Keywords:** advanced knee osteoarthritis, Ahlbäck grade, opening wedge high tibial osteotomy, propensity score matching, unicompartmental knee arthroplasty

## Abstract

**Purpose:**

To compare the clinical outcomes between opening wedge high tibial osteotomy (OWHTO) and unicompartmental knee arthroplasty (UKA) in patients with Ahlbäck Grade 3 medial compartmental knee osteoarthritis (OA) using a propensity score matching (PSM) analysis.

**Methods:**

This retrospective study included all OWHTO and UKA procedures performed between 2016 and 2021 at a single institution. Inclusion criteria were patients diagnosed with medial knee OA, specifically Ahlbäck Grade 3 arthritis. Clinical outcomes were assessed using the Knee Injury and Osteoarthritis Outcome Score (KOOS). Radiographic parameters included hip–knee–ankle (HKA), medial proximal tibial angle (MPTA), lateral distal femoral angle (LDFA) and joint line convergence angle (JLCA). Primary outcomes included improvement in clinical scores at 3, 12 and 24 months post‐operatively. Secondary outcomes included radiographic parameters, complication rates and re‐intervention rates. One‐to‐one PSM was conducted based on gender, age and preoperative KOOS pain scores.

**Results:**

After evaluating eligibility using PSM, a total of 50 knees in the UKA group and 50 knees in the OWHTO group were included. There was no significant difference between groups in preoperative overall KOOS, but the UKA group had better overall KOOS at 3 months (*p* < 0.001). However, the overall KOOS at 12 and 24 months were superior in the OWHTO group compared to the UKA group (12 months; 84.6 ± 4.9 vs. 86.4 ± 2.9, *p* = 0.022, 24 months; 84.9 ± 5.3 vs. 87.0 ± 3.7, *p* = 0.022). As for post‐operative radiological parameters, HKA, MPTA, LDFA and JLCA were higher in the OWHTO group at 24 months post‐operatively. No significant differences were noted in the rates of complication or re‐intervention between groups.

**Conclusions:**

OWHTO can provide outcomes equal to or better than UKA at 24 months post‐operatively in patients with advanced knee OA.

**Level of Evidence:**

Level Ⅲ, Prospective designed retrospective comparative study.

**Abbreviations**
ADLs: activities of daily living
BMI: body mass index
DAIR: debridement, antibiotics and implant retention
HKA: hip–knee–ankle
HTO: high tibial osteotomy
JLCA: joint line convergence angle
JLO: joint line obliquity
KOOS: Knee Injury and Osteoarthritis Outcome Score
LDFA: lateral distal femoral angle
MPTA: medial proximal tibial angle
OA: osteoarthritis
OWHTO: opening wedge high tibial osteotomy
PSM: propensity score matching
QOL: quality of life
ROM: range of motion
TKA: total knee arthroplasty
UKA: unicompartmental knee arthroplasty

## INTRODUCTION

High tibial osteotomy (HTO) and unicompartmental knee arthroplasty (UKA) are well‐established surgical procedures to alleviate symptoms and improve function in patients with medial knee osteoarthritis (OA) [5, 17, 31, 40]. Conventionally, HTO has been preferred for younger patients with less severe OA, while UKA has been preferred for older patients with more advanced OA [5, 18, 21]. However, recent studies have shown promising outcomes after opening wedge HTO (OWHTO), even in patients with severe knee OA [23, 31], suggesting that OWHTO may be feasible across a broader range of disease severity. A number of studies to date have compared the clinical outcomes after HTO and UKA. Since the conventional indication for UKA is limited to anteromedial compartment bone‐on‐bone OA [9, 32], previous studies may contain potential heterogeneity regarding study population characteristics. Therefore, this study focused on patients with Ahlbäck Grade 3 arthritis, which is considered an advanced stage of OA, and aimed to compare the outcomes of OWHTO and UKA using propensity score matching (PSM) to control confounding variables. It was hypothesized that with appropriate patient selection and advanced surgical techniques, OWHTO can provide outcomes comparable to UKA in this specific patient cohort.

## METHODS

### Study population and design

**Figure 1:**
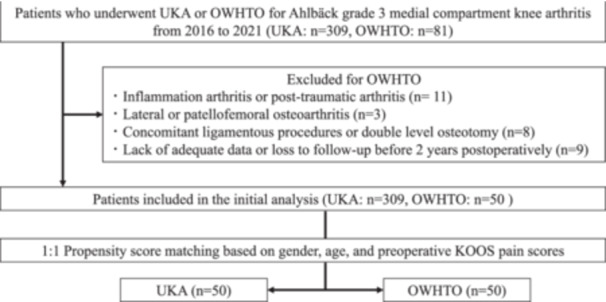
Flowchart of patient selection process. KOOS, Knee Injury and Osteoarthritis Outcome Score; OWHTO, opening wedge high tibial osteotomy; UKA, unicompartmental knee arthroplasty.

This is a retrospective study utilizing prospectively collected data from a single tertiary care institution performing more than 100 UKA and 100 HTO every year. The study period includes all OWHTO and UKA procedures performed between January 2016 and April 2021. Inclusion criteria were patients diagnosed with isolated medial knee OA without apparent ligamentous instability, specifically Ahlbäck Grade 3 arthritis diagnosed two times by two independent senior radiologists on preoperative radiographs in case of non‐conclusive classification between radiologists; a third rating was performed and patients not evaluated as Ahlbäck 3 were excluded from the study [1]. Other exclusion criteria included patients with inflammatory arthritis such as rheumatoid arthritis, post‐traumatic arthritis, significant lateral or patellofemoral compartment OA, and those who underwent concomitant ligamentous procedures or double‐level osteotomies. The process of patient selection is shown in Figure 1. This study was approved by the Institutional Review Board of our institution (PADS21‐151_dgr) and written informed consent was obtained from all the patients.

### Data collection

Demographic and clinical data, including age, gender and body mass index (BMI), were collected for analysis. Post‐operative follow‐up was performed at 3, 12 and 24 months, and clinical outcomes were assessed using the Knee Injury and Osteoarthritis Outcome Score (KOOS). Radiographic parameters such as hip–knee–ankle (HKA), mechanical medial proximal tibial angle (MPTA), mechanical lateral distal femoral angle (LDFA) and joint line convergence angle (JLCA), were evaluated preoperatively and at 24 months. Except in cases of plate removal, patients who underwent revision surgery after OWHTO were excluded from the analysis of subsequent clinical outcomes.

### Propensity score matching

PSM was performed to reduce the effect of selection bias and confounding variables. Patients were matched 1:1 based on gender, age and preoperative KOOS pain scores using a greedy matching algorithm with a calliper width of 0.2 standard deviations of the logit of the propensity score. This approach ensures comparability between the HTO and UKA groups in terms of baseline characteristics.

### Surgical options and procedures

Indication for surgery was based on the radiological assessment of the overall lower limb alignment and bone/joint geometry. As this study included patients with Ahlbäck Grade 3 arthritis who had an intra‐articular deformity, OWHTO was preferred for patients who had both an intra‐articular deformity and an extra‐articular tibial deformity. Meanwhile, UKA was preferred for patients with predominantly intra‐articular deformities, whether or not they had a slight extra‐articular deformity. Both surgeries were performed by two senior surgeons (MO and JA) under general or spinal anaesthesia. The OWHTO procedure involved a medial opening wedge biplanar osteotomy. Preoperative radiological evaluation and surgical planning were conducted on a weight‐bearing long‐leg radiograph. OWHTO was indicated for patients with an isolated tibial deformity with an MPTA of less than 85° and symptomatic intra‐articular pain in the medial compartment. The weight‐bearing line was planned so that the mechanical axis shifted to the lateral tibial spine. Wedge size was determined preoperatively using the method of Miniaci et al [21]. The transverse and ascending biplanar cuts were made to provide rotational stability. A locking plate system (Activmotion® plate, Newclip Technics) with allogenic bone grafting was used for fixation in all cases. Meanwhile, UKA was performed using a fixed‐bearing prosthesis (Persona Partial Knee, Zimmer‐Biomet) through a medial parapatellar approach in all cases. The goal of this procedure was to replace the medial compartment with the knee in slight varus to minimize the risk of lateral compartment wear.

### Post‐operative rehabilitation

Both procedures allowed for immediate weight‐bearing and post‐operative rehabilitation including range of motion exercise (ROM) as tolerated. Especially for OWHTO, if a lateral hinge fracture was identified intraoperatively, non‐weight‐bearing or toe‐touch weight‐bearing was recommended, depending on the type of lateral hinge fracture [33]. Post‐operatively, low‐molecular‐weight heparin was used for thromboprophylaxis for 30 days.

### Outcomes and follow‐up

Primary outcomes included improved KOOS at 3, 12 and 24 months post‐operatively. Secondary outcomes included radiographic parameters, complication rate, and re‐intervention rate. All events of complication and re‐intervention were recorded until final follow‐up. Persistent post‐operative pain, defined as daily pain refractory to medical treatment without significant objective findings, was included in the complication analysis. To minimize bias, data were collected by independent assessors.

### Statistical analysis

All analyses were performed using SPSS Statistics version 28.0 (IBM‐SPSS). The normality of data distribution was assessed using the Shapiro–Wilk test. Continuous variables were reported as mean ± standard deviation and compared using Student's *t* test or the Mann–Whitney *U* test, as appropriate. Categorical variables were compared using the chi‐squared test or Fischer's exact test. Statistical significance was set at *p* < 0.05.

Measurement accuracy was assessed using the intraclass correlation coefficient to indicate intra‐ and interobserver reliability. Two independent assessors reviewed all preoperative and post‐operative radiographs two times in a blinded fashion at 3‐week measurement intervals of non‐conclusive classification between radiologists; a third rating was performed. All intraclass and interclass correlation coefficients were >0.74.

The minimal clinically important difference (MCID) for KOOS has been previously reported in the setting of OWHTO [14]. A power analysis conducted prior to the study's initiation determined that a sample size of 46 patients per group would be sufficient to detect the MCID in KOOS subscores between different time points and/or patient groups, with a statistical power of 80%.

## RESULTS

**Figure 2:**
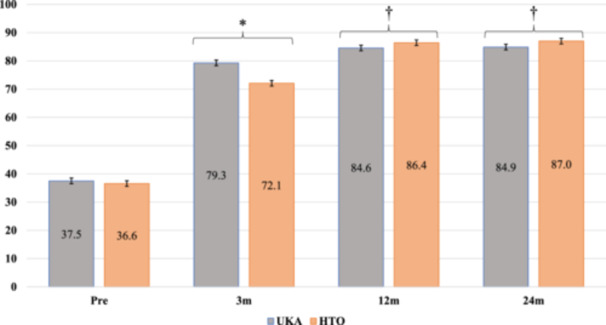
Comparison of perioperative overall KOOS between UKA and OWHTO. *Significantly higher value in the UKA group than in the HTO group. †Significantly higher value in the HTO group than in the UKA group. HTO, high tibial osteotomy; KOOS, Knee Injury and Osteoarthritis Outcome Score; OWHTO, opening wedge high tibial osteotomy; UKA, unicompartmental knee arthroplasty.

**Figure 3:**
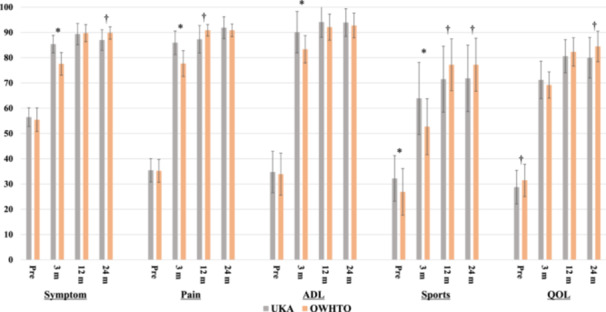
Comparison of perioperative KOOS subscore between UKA and OWHTO. *Significantly higher value in the UKA group than in the HTO group. †Significantly higher value in the HTO group than in the UKA group. HTO, high tibial osteotomy; KOOS, Knee Injury and Osteoarthritis Outcome Score; OWHTO, opening wedge high tibial osteotomy; UKA, unicompartmental knee arthroplasty.

After evaluating eligibility with a 1:1 PSM, a total of 50 knees in the UKA group and 50 knees in the OWHTO group were included in the study. There were no significant differences in patient demographics such as age, gender, BMI or follow‐up period between the two groups (Table 1 **)**. Although there was no significant difference in overall preoperative KOOS between groups (37.5 ± 3.3 vs. 36.6 ± 3.7, *p* = 0.179), the UKA group had better KOOS overall at 3 months (79.3 ± 5.4 vs.72.1 ± 3.8, *p* < 0.001). However, KOOS at 12 and 24 months were superior in the OWHTO group (12 months; 84.6 ± 4.9 vs. 86.4 ± 2.9, *p* = 0.022, 24 months; 84.9 ± 5.3 vs. 87.0 ± 3.7, *p* = 0.022) (Figure 2). Regarding the KOOS subscore, each subscore was significantly higher in the UKA group at 3 months, except for quality of life (QOL). However, KOOS pain and sports/rec scores at 12 months were significantly higher for OWHTO. In addition, KOOS symptoms, sports/rec and QOL scores were also significantly higher in OWHTO at 24 months (Figure 3, Table S1). A comparison of preoperative radiological parameters between the two groups showed significant differences in preoperative HKA, LDFA and JLCA, suggesting less preoperative varus with a greater JLCA in the UKA group. As for post‐operative radiological parameters, the OWHTO group showed higher HKA, MPTA, LDFA and JLCA values at 24 months post‐operatively than the UKA group (Table 2). Three patients in the UKA group underwent conversion total knee arthroplasty (TKA), but no significant differences were noted in the rates of complication or re‐intervention between groups (Table 3). The reason for conversion to TKA was residual pain in two cases and a tibial plateau fracture due to post‐operative apparent trauma in one case.

**Table 1:** Clinical characteristics.

	UKA (*n* = 50)	OWHTO (*n* = 50)	*p*
Age (years)	55.4 ± 4.4 (50–66)	54.4 ± 3.4 (50–63)	0.227
Gender (female, %)	21 (42%)	21 (42%)	1
BMI	28 ± 1.4 (25.4–31.5)	29 ± 1.5 (21.5–31.2)	0.727
Follow‐up period (months)	30.9 ± 6.5 (24–42)	29.3 ± 4.1 (24–41)	0.167

*Note*: Values are expressed as means and standard deviations, with ranges in parentheses.

Abbreviations: BMI, body mass index; OWHTO, opening wedge high tibial osteotomy; UKA, unicompartmental knee arthroplasty.

**Table 2:** Comparison of radiological parameters between UKA and OWHTO.

		UKA	OWHTO	*p*
HKA	Preoperative	**171.7** ± **3.1**	**170.3** ± **3.3**	**0.032**
	24 months	**178.2** ± **3.3**	**181.2** ± **2.8**	**<0.001**
MPTA	Preoperative	84.9 ± 1.9	84.1 ± 2.4	0.076
	24 months	**86.1** ± **1.7**	**91.3** ± **1.6**	**<0.001**
LDFA	Preoperative	**88.4** ± **1.2**	**89.1** ± **1.5**	**0.021**
	24 months	**88.5** ± **1.2**	**89.0** ± **1.6**	**0.043**
JLCA	Preoperative	**5.2** ± **1.9**	**4.2** ± **1.6**	**0.007**
	24 months	**−0.2** ± **1.4**	**2.3** ± **4.1**	**<0.001**

*Note*: Values are expressed as means and standard deviations. Bold values indicate a statistically significant difference.

Abbreviations: HKA, hip–knee–ankle; JLCA, joint line convergence angle; LDFA, lateral distal femoral angle; MPTA, medial proximal tibial angle; OWHTO, opening wedge high tibial osteotomy; UKA, unicompartmental knee arthroplasty.

**Table 3:** Comparison of complication rates and re‐intervention rates between UKA and OWHTO.

	UKA (*n* = 50)	OWHTO (*n* = 50)	*p*
Complication			
Overall (%)	6 (12%)	7 (14%)	0.766
Intraoperative or post‐operative fracture (%)	0 (0%)	2 (4%)	
Infection (%)	1 (2%)	1 (2%)	
Persistent knee pain (%)	5 (10%)	0 (0%)	
Implant related symptoms (%)	0 (0%)	4 (8%)	
Re‐intervention			
Overall (%)	4 (8%)	5 (10%)	0.727
DAIR (%)	1 (2%)	1 (2%)	
TKA conversion (%)	3 (6%)	0 (0%)	
Implant removal (%)	N.A	4 (8%)	

Abbreviations: DAIR, debridement, antibiotics and implant retention; OWHTO, opening wedge high tibial osteotomy; TKA, total knee arthroplasty; UKA, unicompartmental knee arthroplasty.

## DISCUSSION

The main finding of this study was that in this specific cohort of patients with Ahlbäck Grade 3 OA, OWHTO can provide clinical outcomes comparable to or better than UKA at 24 months post‐operatively. In addition, there were no significant differences in the incidence of complications or re‐intervention between the two groups. Therefore, the hypothesis of this study was confirmed.

A number of studies have compared clinical outcomes between UKA and HTO [5, 12, 13, 25, 37, 41, 42, 43]. According to recent review articles, UKA has resulted in fewer complications, lower revision rates, and better post‐operative pain relief, while HTO may lead to better ROM and higher post‐operative activity [4, 34, 43]. In the last decade, surgical techniques and management for knee osteotomies have advanced significantly, and extensive knowledge has highlighted the importance of the JLCA and joint line obliquity (JLO) as a prognostic factor [2, 29, 30]. However, these recent advancements have necessitated a reevaluation of the treatment paradigms for OWHTO. In addition, there may be potential drawbacks such as population heterogeneity in the literature. Since UKA is preferred over bone‐on‐bone OA in elderly patients [9, 32], the comparison of clinical outcomes between UKA and HTO should be based on the same degree of preoperative OA severity and population age. Furthermore, radiological classification of osteoarthritic knees with bone‐on‐bone OA, such as Kellgren–Lawrence Grade 4, can be further subdivided into Ahlbäck Grades 2–5 [1]. Therefore, we applied the Ahlbäck classification system, which may be more effective in accurately assessing the advanced stages of OA, and specifically included Ahlbäck Grade 3 OA in this study.

To date, there are conflicting findings regarding outcomes after HTO for advanced knee OA. Several studies have reported that advanced knee OA with severe chondral wear may pose a risk of clinical failure [16, 39]. Meanwhile, recent studies have shown that even patients with advanced medial OA can achieve promising results following OWHTO [23, 31]. This study also showed better clinical outcomes after OWHTO than after UKA at 12 and 24 months, even though HKA was slightly corrected to a valgus alignment. Possible reasons for the superior outcomes after OWHTO may be due to recent developments in the OWHTO technique, such as the use of a hinge protection wire, the estimation of perioperative JLCA changes, and limited surgical indication [6, 8, 29, 30]. In addition, when we assumed the post‐operative MPTA exceeded 94°, OWHTO was avoided to prevent post‐operative iatrogenic JLO, indicating that OWHTO was mainly performed in patients with extra‐articular tibial deformity.

Previous studies have shown that post‐operative recovery could be achieved more quickly with UKA than HTO, especially in the first post‐operative year [12, 19, 35]. In this study, OWHTO outperformed UKA in clinical outcomes from 12 to 24 months, although similar trends were observed at 3 months. As for the KOOS subscore, this study found superior outcomes for OWHTO with regard to symptoms, sports and recreation, and QOL at 24 months. Several studies have demonstrated that UKA could provide higher activity than HTO [10, 22]; however, these studies may include potential shortcomings of closing wedge HTO or a conventional post‐operative protocol, such as weight‐bearing restriction for 4–6 weeks. On the contrary, recent studies have reported higher activity levels were achieved after OWHTO than UKA [3, 36]. The present study also showed similar results after PSM analysis to recent findings addressing OWHTO in patients with advanced OA.

Few studies comparing UKA and OWHTO have investigated detailed information on the coronal alignment of the lower limb using whole‐leg radiographs [41]. Teo et al. conducted a comparative study of HTO and UKA in patients with Kellgren–Lawrence Grade 4 using PSM analysis, which showed detailed radiographic parameters. They also reported comparable outcomes between the two procedures, even though the HTO group had a mean post‐operative HKA of 0.6° valgus. Consistent with the findings of Teo et al., this study also demonstrated that even with a mean post‐operative HKA of 1.2°, satisfactory clinical outcomes were achieved after OWHTO. Conventionally, surgeons have aimed to correct patients with varus medial arthritic knees to an increased valgus alignment equivalent to HKA 3–6° [7, 31]. In advanced knee OA, the large JLCA due to intra‐articular deformity and soft tissue laxity [28] presents a risk of over‐correction when surgeons aim for the conventional valgus alignment without a specific method to estimate perioperative JLCA change [7, 24, 33]. Overcorrection after OWHTO may result in inferior patient satisfaction [26]; however, when treating advanced OA after OWHTO, the risk of long‐term progression of recurrent varus deformity should also be considered [27]. Therefore, a longer follow‐up period is needed to determine the impact of optimal post‐operative alignment on long‐term clinical outcomes.

With regard to complications and re‐interventions, there was no significant difference in complication rate between groups in the present study, but the revision rate after UKA was 6%. Although a higher revision rate was observed in this study despite the short follow‐up period, previous registry studies have also shown a high revision rate in younger patients, especially those younger than 55 years old [15]. Jeschke et al. reported that approximately 20% of patients younger than 55 underwent revision surgery within 5 years after their initial treatment. Therefore, the younger age of our UKA population (55.4 years) may have influenced these results. In addition, the mean post‐operative JLCA in this cohort was −0.2°, suggesting that overcorrection may have occurred after UKA in this study, in contrast to the surgical concept of intra‐articular correction and resurfacing in UKA. If a patient is overcorrected after UKA, progressive OA change may occur in the lateral compartment [11, 20, 38]. These factors may also have influenced the findings on the revision rate of this study.

There are several limitations in the design and content of this study. First, this was a single‐centre, retrospective comparative study with short‐term outcomes. Nevertheless, to reduce the selection bias present in previous literature, this study employed PSM analysis. Second, there were slight but significant differences in the preoperative radiographic parameters, such as HKA, LDFA and JLCA, even though PSM analysis was performed. Compared to OWHTO, UKA was primarily indicated for patients with intra‐articular deformities. Although the study population was limited to Ahlbäck Grade 3 OA, selection bias may still exist. However, these differences may be due to the concept of each surgical procedure. Third, the relatively high revision rate after UKA was noted in this study. This may affect clinical outcomes after UKA, despite the exclusion of patients who required additional intervention to assess clinical outcomes. In addition, the present study excluded patients who required revision surgery except for the patients with plate removal. However, this may introduce the selection bias by excluding patients with worse outcomes, overestimating the favourable results of OWHTO and underestimating its potential complications. Fourth, the relationships between patient‐reported outcomes and radiological outcomes were not evaluated in the present study. Finally, the present study was aimed at comparing clinical outcomes up to 24 months between UKA and OWHTO. Therefore, a longer follow‐up period would be required to assess the effect of each procedure on long‐term clinical outcomes.

Despite its limitations, this study is significant because it compared clinical and radiological outcomes between two groups with similar backgrounds and appropriate patient selection. Further prospective and longer studies with larger sample sizes and appropriate patient selection are needed to determine the optimal strategies for patients with advanced‐stage knee OA.

## CONCLUSIONS

OWHTO can provide outcomes comparable to or better than UKA at 24 months post‐operatively in patients with advanced knee OA. In addition, there were no significant differences in the incidence of complications or re‐intervention between the two groups.

## AUTHOR CONTRIBUTIONS

All authors have contributed to the design, content and writing of the manuscript. All authors read and approved the final manuscript.

## CONFLICT OF INTEREST STATEMENT

Matthieu Ollivier has received consulting fees from Newclip Technics, Arthrex and Stryker. Jean‐Noël Argenson has received consulting fees from Zimmer‐Biomet. The remaining authors declare no conflict of interest.

## ETHICS STATEMENT

Ethical approval for this study was obtained from the Institutional Review Board in our institution (PADS21‐151_dgr). Informed consent was obtained from all individual participants included in the study.

## Supporting information

Supporting information.

## Data Availability

The data that support the findings of this study are available from the corresponding author upon reasonable request.

## References

[1] Ahlback, S. (1968) Osteoarthrosis of the knee. A radiographic investigation. Acta Radiologica: Diagnosis (Stockh), 277, 7–72. 5706059

[2] Behrendt, P. , Akoto, R. , Bartels, I. , Thürig, G. , Fahlbusch, H. , Korthaus, A. et al. (2023) Preoperative joint line convergence angle correction is a key factor in optimising accuracy in varus knee correction osteotomy. Knee Surgery, Sports Traumatology, Arthroscopy, 31, 1583–1592. Available from: 10.1007/s00167-022-07092-2 PMC10049955 35994079

[3] Belsey, J. , Yasen, S.K. , Jobson, S. , Faulkner, J. & Wilson, A.J. (2021) Return to physical activity after high tibial osteotomy or unicompartmental knee arthroplasty: a systematic review and pooling data analysis. The American Journal of Sports Medicine, 49, 1372–1380. Available from: 10.1177/0363546520948861 32960075 PMC8020302

[4] Cao, Z. , Mai, X. , Wang, J. , Feng, E. & Huang, Y. (2018) Unicompartmental knee arthroplasty vs high tibial osteotomy for knee osteoarthritis: a systematic review and meta‐analysis. The Journal of Arthroplasty, 33, 952–959. Available from: 10.1016/j.arth.2017.10.025 29203354

[5] Debopadhaya, S. , Acosta, E. , & Ortiz 3rd D. (2024) Trends and outcomes in the surgical management of young adults with knee osteoarthritis using high tibial osteotomy and unicompartmental knee arthroplasty. Archives of Orthopaedic and Trauma Surgery, 144, 3995–4002. Available from: 10.1007/s00402-024-05362-x 38771360

[6] Dessyn, E. , Sharma, A. , Donnez, M. , Chabrand, P. , Ehlinger, M. , Argenson, J.N. et al. (2020) Adding a protective K‐wire during opening high tibial osteotomy increases lateral hinge resistance to fracture. Knee Surgery, Sports Traumatology, Arthroscopy, 28, 751–758. Available from: 10.1007/s00167-019-05404-7 30783689

[7] Fujisawa, Y. , Masuhara, K. & Shiomi, S. (1979) The effect of high tibial osteotomy on osteoarthritis of the knee. Orthopedic Clinics of North America, 10, 585–608. Available from: 10.1016/S0030-5898(20)30753-7 460834

[8] Gulagaci, F. , Jacquet, C. , Ehlinger, M. , Sharma, A. , Kley, K. , Wilson, A. et al. (2020) A protective hinge wire, intersecting the osteotomy plane, can reduce the occurrence of perioperative hinge fractures in medial opening wedge osteotomy. Knee Surgery, Sports Traumatology, Arthroscopy, 28, 3173–3182. Available from: 10.1007/s00167-019-05806-7 31773202

[9] Hamilton, T.W. , Pandit, H.G. , Jenkins, C. , Mellon, S.J. , Dodd, C.A.F. & Murray, D.W. (2017) Evidence‐based indications for mobile‐bearing unicompartmental knee arthroplasty in a consecutive cohort of thousand knees. The Journal of Arthroplasty, 32, 1779–1785. Available from: 10.1016/j.arth.2016.12.036 28131544

[10] Han, S.B. , Kyung, H.S. , Seo, I.W. & Shin, Y.S. (2017) Better clinical outcomes after unicompartmental knee arthroplasty when comparing with high tibial osteotomy. Medicine, 96, e9268. Available from: 10.1097/MD.0000000000009268 29390376 PMC5815788

[11] Hernigou, P. & Deschamps, G. (2004) Alignment influences wear in the knee after medial unicompartmental arthroplasty. Clinical Orthopaedics and Related Research, 423, 161–165. Available from: 10.1097/01.blo.0000128285.90459.12 15232443

[12] Hoorntje, A. , Pronk, Y. , Brinkman, J.M. , van Geenen, R.C.I. & van Heerwaarden, R.J. (2023) High tibial osteotomy versus unicompartmental knee arthroplasty for Kellgren‐Lawrence grade 3‐4 knee osteoarthritis in younger patients: comparable improvements in patient‐reported outcomes, adjusted for osteoarthritis grade and sex. Knee Surgery, Sports Traumatology, Arthroscopy, 31, 4861–4870. Available from: 10.1007/s00167-023-07526-5 PMC10598142 37572139

[13] Huang, L. , Xu, Y. , Wei, L. , Yuan, G. , Chen, W. , Gao, S. et al. (2022) Unicompartmental knee arthroplasty is superior to high tibial osteotomy for the treatment of medial unicompartmental osteoarthritis: a systematic review and meta‐analysis. Medicine, 101, e29576. Available from: 10.1097/MD.0000000000029576 35905249 PMC9333480

[14] Jacquet, C. , Pioger, C. , Khakha, R. , Steltzlen, C. , Kley, K. , Pujol, N. et al. (2021) Evaluation of the “Minimal Clinically Important Difference” (MCID) of the KOOS, KSS and SF‐12 scores after open‐wedge high tibial osteotomy. Knee Surgery, Sports Traumatology, Arthroscopy, 29, 820–826. Available from: 10.1007/s00167-020-06026-0 32342141

[15] Jeschke, E. , Gehrke, T. , Günster, C. , Hassenpflug, J. , Malzahn, J. , Niethard, F.U. et al. (2016) Five‐year survival of 20,946 unicondylar knee replacements and patient risk factors for failure: an analysis of german insurance data. Journal of Bone and Joint Surgery, 98, 1691–1698. Available from: 10.2106/JBJS.15.01060 27869619

[16] Jin, C. , Song, E.K. , Santoso, A. , Ingale, P.S. , Choi, I.S. & Seon, J.K. (2020) Survival and risk factor analysis of medial open wedge high tibial osteotomy for unicompartment knee osteoarthritis. Arthroscopy: The Journal of Arthroscopic & Related Surgery, 36, 535–543. Available from: 10.1016/j.arthro.2019.08.040 31901391

[17] Jin, Q.H. , Lee, W.G. , Song, E.K. , Jin, C. & Seon, J.K. (2021) Comparison of long‐term survival analysis between open‐wedge high tibial osteotomy and unicompartmental knee arthroplasty. The Journal of Arthroplasty, 36, 1562–1567.e1. Available from: 10.1016/j.arth.2020.11.008 33261999

[18] Kawata, M. , Sasabuchi, Y. , Inui, H. , Taketomi, S. , Matsui, H. , Fushimi, K. et al. (2017) Annual trends in knee arthroplasty and tibial osteotomy: analysis of a national database in Japan. The Knee, 24, 1198–1205. Available from: 10.1016/j.knee.2017.06.005 28797877

[19] Kim, M.S. , Koh, I.J. , Sohn, S. , Jeong, J.H. & In, Y. (2019) Unicompartmental knee arthroplasty is superior to high tibial osteotomy in post‐operative recovery and participation in recreational and sports activities. International Orthopaedics, 43, 2493–2501. Available from: 10.1007/s00264-018-4272-5 30565177

[20] Kim, S.J. , Bae, J.H. & Lim, H.C. (2012) Factors affecting the postoperative limb alignment and clinical outcome after Oxford unicompartmental knee arthroplasty. The Journal of Arthroplasty, 27, 1210–1215. Available from: 10.1016/j.arth.2011.12.011 22285234

[21] Koh, I.J. , Kim, M.W. , Kim, J.H. , Han, S.Y. & In, Y. (2015) Trends in high tibial osteotomy and knee arthroplasty utilizations and demographics in Korea from 2009 to 2013. The Journal of Arthroplasty, 30, 939–944. Available from: 10.1016/j.arth.2015.01.002 25639855

[22] Krych, A.J. , Reardon, P. , Sousa, P. , Pareek, A. , Stuart, M. & Pagnano, M. (2017) Unicompartmental knee arthroplasty provides higher activity and durability than valgus‐producing proximal tibial osteotomy at 5 to 7 years. Journal of Bone and Joint Surgery, 99, 113–122. Available from: 10.2106/JBJS.15.01031 28099301

[23] Lee, B.S. , Kim, T.H. , Bin, S.I. , Kim, J.M. & Kim, H. (2021) Clinicoradiologic outcomes of medial open‐wedge high‐tibial osteotomy are equivalent in bone‐on‐bone and non‐bone‐on‐bone medial osteoarthritis. Arthroscopy: The Journal of Arthroscopic & Related Surgery, 37, 638–644. Available from: 10.1016/j.arthro.2020.09.033 32998040

[24] Lee, D. , Wang, J.H. , Won, Y. , Min, Y.K. , Jaiswal, S. , Lee, B.H. et al. (2020) Preoperative latent medial laxity and correction angle are crucial factors for overcorrection in medial open‐wedge high tibial osteotomy. Knee Surgery, Sports Traumatology, Arthroscopy, 28, 1411–1418. Available from: 10.1007/s00167-019-05502-6 30980121

[25] Lee, S.H. , Kim, H.R. , Seo, H.Y. & Seon, J.K. (2022) A comparative study of 21,194 UKAs and 49,270 HTOs for the risk of unanticipated events in mid‐age patients from the national claims data in South Korea. BMC Musculoskeletal Disorders, 23, 127. Available from: 10.1186/s12891-022-05080-8 35135508 PMC8827168

[26] Lee, S.S. , Kim, J.H. , Kim, S. , Jung, E.Y. , Ryu, D.J. , Lee, D.K. et al. (2022) Avoiding overcorrection to increase patient satisfaction after open wedge high tibial osteotomy. The American Journal of Sports Medicine, 50, 2453–2461. Available from: 10.1177/03635465221102144 35722821

[27] Lee, S.S. , Lee, Y.K. , Kim, I.S. , Ryu, D.J. , Jung, E.Y. , Lee, D.K. et al. (2022) Preoperative medial tightness and narrow medial joint space are predictive factors for lower extremity alignment change toward varus after opening‐wedge high tibial osteotomy. Orthopaedic Journal of Sports Medicine, 10, 23259671221119152. Available from: 10.1177/23259671221119152 36062158 PMC9434689

[28] Mabrouk, A. , An, J.S. , Glauco, L. , Jacque, C. , Kley, K. , Sharma, A. et al. (2023) The joint line convergence angle (JLCA) correlates with intra‐articular arthritis. Knee Surgery, Sports Traumatology, Arthroscopy, 31, 5673–5680. Available from: 10.1007/s00167-023-07616-4 37884727

[29] Micicoi, G. , Khakha, R. , Kley, K. , Wilson, A. , Cerciello, S. & Ollivier, M. (2020) Managing intra‐articular deformity in high tibial osteotomy: a narrative review. Journal of Experimental Orthopaedics, 7, 65. Available from: 10.1186/s40634-020-00283-1 32902758 PMC7481321

[30] Nakayama, H. , Schröter, S. , Yamamoto, C. , Iseki, T. , Kanto, R. , Kurosaka, K. et al. (2018) Large correction in opening wedge high tibial osteotomy with resultant joint‐line obliquity induces excessive shear stress on the articular cartilage. Knee Surgery, Sports Traumatology, Arthroscopy, 26, 1873–1878. Available from: 10.1007/s00167-017-4680-x 28831525

[31] Ollivier, B. , Berger, P. , Depuydt, C. & Vandenneucker, H. (2021) Good long‐term survival and patient‐reported outcomes after high tibial osteotomy for medial compartment osteoarthritis. Knee Surgery, Sports Traumatology, Arthroscopy, 29, 3569–3584. Available from: 10.1007/s00167-020-06262-4 32909057

[32] Pandit, H. , Gulati, A. , Jenkins, C. , Barker, K. , Price, A.J. , Dodd, C.A.F. et al. (2011) Unicompartmental knee replacement for patients with partial thickness cartilage loss in the affected compartment. The Knee, 18, 168–171. Available from: 10.1016/j.knee.2010.05.003 20627734

[33] Park, J.G. , Kim, J.M. , Lee, B.S. , Lee, S.M. , Kwon, O.J. & Bin, S.I. (2020) Increased preoperative medial and lateral laxity is a predictor of overcorrection in open wedge high tibial osteotomy. Knee Surgery, Sports Traumatology, Arthroscopy, 28, 3164–3172. Available from: 10.1007/s00167-019-05805-8 31781797

[34] Ping, H. , Wen, J. , Liu, Y. , Li, H. , Wang, X. , Kong, X. et al. (2022) Unicompartmental knee arthroplasty is associated with lower pain levels but inferior range of motion, compared with high tibial osteotomy: a systematic overview of meta‐analyses. Journal of Orthopaedic Surgery and Research, 17, 425. Available from: 10.1186/s13018-022-03319-7 36153554 PMC9509560

[35] Ryu, S.M. , Park, J.W. , Na, H.D. & Shon, O.J. (2018) High tibial osteotomy versus unicompartmental knee arthroplasty for medial compartment arthrosis with kissing lesions in relatively young patients. Knee Surgery and Related Research, 30, 17–22. Available from: 10.5792/ksrr.17.006 29298462 PMC5853178

[36] Screpis, D. , Piovan, G. , Baldini, M. , Amarossi, A. , Natali, S. , Iacono, V. et al. (2023) Higher activity level after opening wedge high tibial osteotomy compared to medial unicompartimental knee arthroplasty in a selected cohort of advanced age: a propensity score‐matched analysis. The Knee, 40, 183–191. Available from: 10.1016/j.knee.2022.11.006 36470195

[37] Siren, J. , Rämö, L. , Rantasalo, M. , Komulainen, O. , Skants, N. , Reito, A. et al. (2023) Unicompartmental knee arthroplasty vs. high tibial osteotomy for medial knee osteoarthritis (UNIKORN): a study protocol of a randomized controlled trial. Trials, 24, 256. Available from: 10.1186/s13063-023-07263-7 37016454 PMC10074655

[38] Slaven, S.E. , Cody, J.P. , Sershon, R.A. , Ho, H. , Hopper Jr., R.H. & Fricka, K.B. (2020) The impact of coronal alignment on revision in medial fixed‐bearing unicompartmental knee arthroplasty. The Journal of Arthroplasty, 35, 353–357. Available from: 10.1016/j.arth.2019.09.038 31668526

[39] Sohn, S. , Koh, I.J. , Kim, M.S. , Kang, B.M. & In, Y. (2020) What factors predict patient dissatisfaction after contemporary medial opening‐wedge high tibial osteotomy? The Journal of Arthroplasty, 35, 318–324. Available from: 10.1016/j.arth.2019.09.026 31630965

[40] Tay, M.L. , Bolam, S.M. , Maxwell, A.R. , Hooper, G.J. , Monk, A.P. & Young, S.W. (2023) Similar survivorship but different revision reasons for uncemented mobile‐bearing and cemented fixed‐bearing medial UKA: a long‐term population‐based cohort study of 2,015 patients. Journal of Bone and Joint Surgery, 105, 755–761. Available from: 10.2106/JBJS.22.00686 36812351

[41] Teo, S.J. , Purnomo, G. , Koh, D.T.S. , Soong, J. , Yeo, W. , Razak, H.R.B.A. et al. (2024) High tibial osteotomy versus unicompartmental knee arthroplasty in advanced medial compartmental knee arthrosis: a comparative study with propensity score matched analysis. The Knee, 49, 116–124. Available from: 10.1016/j.knee.2024.06.003 38909589

[42] Wyatt, F.W. & Al‐Dadah, O. (2024) Unicompartmental knee arthroplasty vs high tibial osteotomy for knee osteoarthritis: a comparison of clinical and radiological outcomes. World Journal of Orthopedics, 15, 444–456. Available from: 10.5312/wjo.v15.i5.444 38835690 PMC11145972

[43] Zhang, B. , Qian, H. , Wu, H. & Yang, X. (2023) Unicompartmental knee arthroplasty versus high tibial osteotomy for medial knee osteoarthritis: a systematic review and meta‐analysis. Journal of Orthopaedic Surgery, 31, 10225536231162829. Available from: 10.1177/10225536231162829 36893443

