# Supplementary material for: Opening wedge high tibial osteotomy yields comparable to superior outcomes to unicompartmental knee arthroplasty at 2 years of follow‐up in patients suffering from Ahlbäck III knee osteoarthritis: A propensity score‐matched analysis
Source: J Exp Orthop. 2024 Dec 2;11(4):e70105. doi: 10.1002/jeo2.70105 (PMC11609990; doi:10.1002/jeo2.70105)
Supplement: Supplementary file 1 — Supporting information. [file JEO2-11-e70105-s001.pptx]

## Slide 1
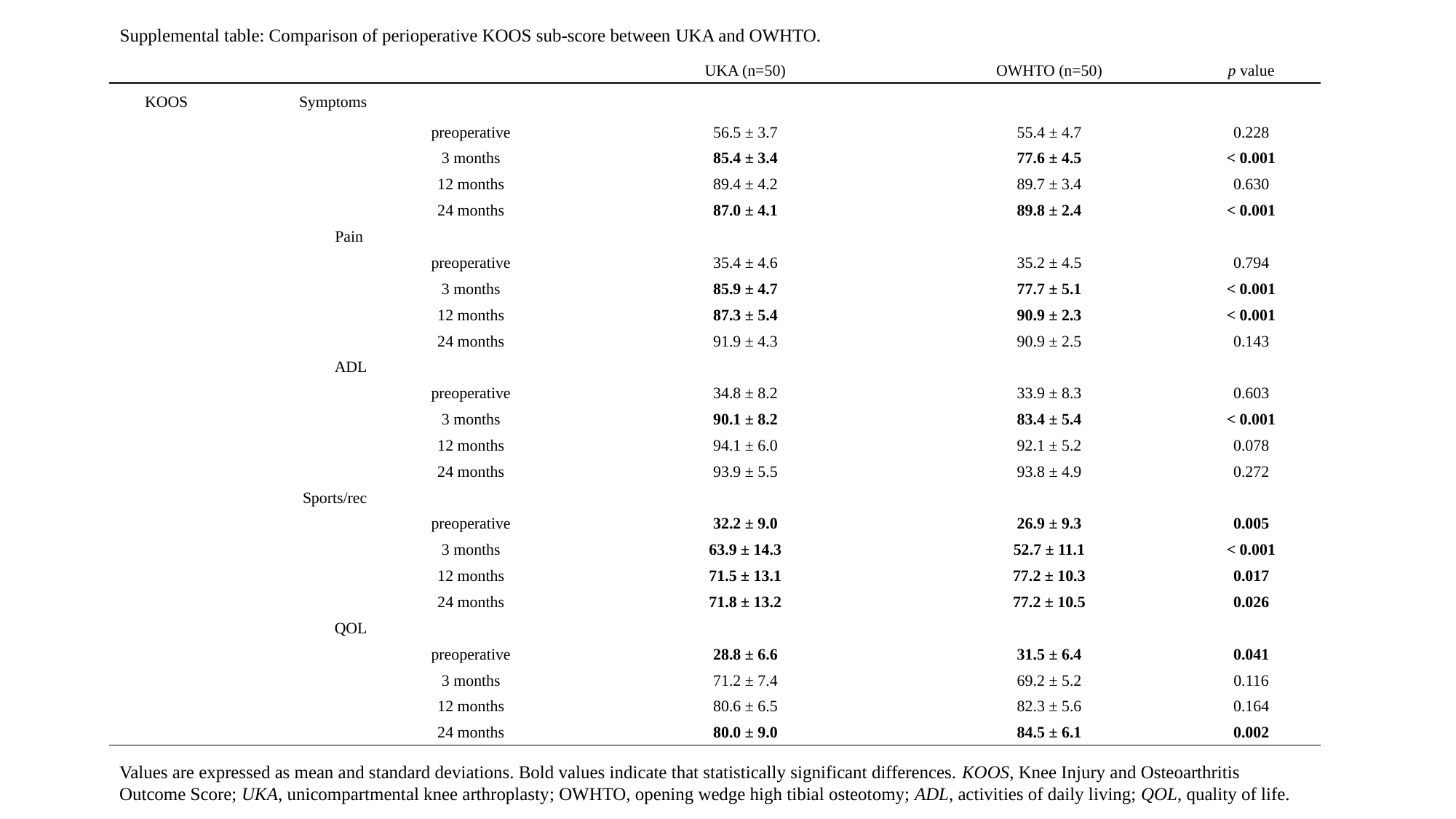

# Supplemental table: Comparison of perioperative KOOS sub-score between UKA and OWHTO.
| | | UKA (n=50) | OWHTO (n=50) | p value |
| --- | --- | --- | --- | --- |
| KOOS　　　　　　 Symptoms | | | | |
| | preoperative | 56.5 ± 3.7 | 55.4 ± 4.7 | 0.228 |
| | 3 months | 85.4 ± 3.4 | 77.6 ± 4.5 | < 0.001 |
| | 12 months | 89.4 ± 4.2 | 89.7 ± 3.4 | 0.630 |
| | 24 months | 87.0 ± 4.1 | 89.8 ± 2.4 | < 0.001 |
| Pain | | | | |
| | preoperative | 35.4 ± 4.6 | 35.2 ± 4.5 | 0.794 |
| | 3 months | 85.9 ± 4.7 | 77.7 ± 5.1 | < 0.001 |
| | 12 months | 87.3 ± 5.4 | 90.9 ± 2.3 | < 0.001 |
| | 24 months | 91.9 ± 4.3 | 90.9 ± 2.5 | 0.143 |
| ADL | | | | |
| | preoperative | 34.8 ± 8.2 | 33.9 ± 8.3 | 0.603 |
| | 3 months | 90.1 ± 8.2 | 83.4 ± 5.4 | < 0.001 |
| | 12 months | 94.1 ± 6.0 | 92.1 ± 5.2 | 0.078 |
| | 24 months | 93.9 ± 5.5 | 93.8 ± 4.9 | 0.272 |
| Sports/rec | | | | |
| | preoperative | 32.2 ± 9.0 | 26.9 ± 9.3 | 0.005 |
| | 3 months | 63.9 ± 14.3 | 52.7 ± 11.1 | < 0.001 |
| | 12 months | 71.5 ± 13.1 | 77.2 ± 10.3 | 0.017 |
| | 24 months | 71.8 ± 13.2 | 77.2 ± 10.5 | 0.026 |
| QOL | | | | |
| | preoperative | 28.8 ± 6.6 | 31.5 ± 6.4 | 0.041 |
| | 3 months | 71.2 ± 7.4 | 69.2 ± 5.2 | 0.116 |
| | 12 months | 80.6 ± 6.5 | 82.3 ± 5.6 | 0.164 |
| | 24 months | 80.0 ± 9.0 | 84.5 ± 6.1 | 0.002 |
Values are expressed as mean and standard deviations. Bold values indicate that statistically significant differences. KOOS, Knee Injury and Osteoarthritis Outcome Score; UKA, unicompartmental knee arthroplasty; OWHTO, opening wedge high tibial osteotomy; ADL, activities of daily living; QOL, quality of life.
